# Automated and Model-Free Bridge Damage Indicators with Simultaneous Multiparameter Modal Anomaly Detection

**DOI:** 10.3390/s20174752

**Published:** 2020-08-22

**Authors:** Thanh T. X. Tran, Ekin Ozer

**Affiliations:** 1 Department of Civil Engineering, Yokohama National University, Yokohama 240-8501, Japan; thanhttx@gmail.com; 2 Department of Civil & Environmental Engineering, University of Strathclyde, Glasgow G1 1XQ, UK

**Keywords:** system identification, structural health monitoring, system realization using information matrix, automated modal analysis, seismic damage assessment, anomaly detection

## Abstract

This paper pursues a simultaneous modal parameter anomaly detection paradigm to structural damage identification inferred from vibration-based structural health monitoring (SHM) sensors, e.g., accelerometers. System Realization Using Information Matrix (SRIM) method is performed in short duration sweeping time windows for identification of state matrices, and then, modal parameters with enhanced automation. Stable modal poles collected from stability diagrams are clustered and fed into the Gaussian distribution-based anomaly detection platform. Different anomaly thresholds are examined both on frequency and damping ratio terms taking two testbed bridge structures as application means, and simplistic Boolean Operators are performed to merge univariate anomalies. The first bridge is a reinforced concrete bridge subjected to incremental damage through a series of seismic shake table experiments conducted at the University of Nevada, Reno. The second bridge is a steel arch structure at Columbia University Morningside Campus, which reflects no damage throughout the measurements, unlike the first one. Two large-scale implementations indicate the realistic performance of automated modal analysis and anomaly recognition with minimal human intervention in terms of parameter extraction and learning supervision. Anomaly detection performance, presented in this paper, shows variation according to the designated thresholds, and hence, the information retrieval metrics being considered. The methodology is well-fitted to SHM problems which require sole data-driven, scalable, and fully autonomous perspectives.

## 1. Introduction

Structural Health Monitoring (SHM), with a pattern recognition perspective, seeks the ability to detect abnormal changes corresponding to structural damage at the earliest stages possible [1,2,3]. Sensor-model combination infers damage-sensitive features which can trigger the necessary alerts before a catastrophic event occurs [4,5,6,7]. During or right-after extreme force conditions, e.g., earthquakes, structural changes may be spotted via sensor data in real-time to direct essential precautions and preventive actions. Aging infrastructure also requires similar strategies to identify damage indicator anomalies due to failure and prioritize retrofit strategies where necessary.

A large percentage of the current literature successfully addresses SHM methods depending on analytical models and a fine-tuned comparative scenario using prior information/test data for undamaged/damage state comparison [8,9,10]. The methods often require advanced engineering intervention during the implementation process, e.g., model development and updating. Moreover, the requirement of the pre-event data may have an unclear line with the post-event in many cases. Hence, the development of entirely model-free damage detection methods using measurement data receives much attention recently [11,12,13,14]. Compared with the model-based methods, the model-free ones can be performed more automatically and time efficiently.

Moreover, together with the rapid development of advanced microelectromechanical system (MEMS) technologies in recent decades, more reliable and affordable multisensory data are available [15]. Therefore, heterogeneous and ubiquitous data can compensate for the uncertainties related to infrastructures—leaving model-driven perspective out in damage detection—if a proper framework is established with correct usage of damage-sensitive features. It is essential to note that model-free approaches have limitations in terms of depth of SHM knowledge and can improve the information level with complementary sources (e.g., model updating).

Anomaly detection, also referred to as outlier detection, can help to find abnormal health conditions/changes given a dataset during the operational period of structures and hence can detect structural damages. Anomaly detection is typically an unsupervised machine learning application to detect damage in SHM, ideally in a model-free fashion. An anomaly could be caused by changes related to the structure itself, operational forces acting on the structures, or environmental effects (temperature, wind, humidity, and more) [16,17]. Likewise, sensor deficiencies trigger anomalies, and system identification algorithms can influence the variation in dynamic parameters. In other words, an anomaly may not always imply damage and can be associated with dysfunctional data, environmental/operational effects, or even depend on the identification methods being used. Only if these secondary aspects are treated with necessary processing schemes where applicable, the damage indication can be associated with the anomaly.

It should be noted that the aforementioned secondary aspects become more influential in the field implementations due to uncontrolled environmental/operational conditions and more. Moreover, numerous detection evaluator metrics, which are later on expressed in this paper, cannot apply to real-life examples due to lack of damage data before specific events. Nevertheless, in this paper, the presented cases address simultaneous indicators, including damaged and nondamaged laboratory and field scenarios. One standard method to damage detection is using vibration data to track any changes in structural properties, e.g., stiffness, mass, and dissipated energy. This tracking can be done using damage-sensitive modal features such as natural frequency, damping ratio, and mode shapes [18].

Anomaly detection procedure based on modal tracking using observation data can be divided into three major stages. The first stage includes real-time data acquisition and signal processing, the second stage stands for modal parameter estimation and tracking, and the final stage consists of anomaly detection using the identified modal parameters. This study concentrates on the second and third steps, considering the time evolution of observation data in a simultaneous setting. Implementation of these steps relies on automation, yet high accuracy, within limited lengths of data.

Simultaneous identification focuses not only on autonomous mechanisms but also on short-time procession, prioritizing computational speed, parallelization, and hardware requirements. In this study, automation aspects are scoped, covering operational modal analysis and anomaly detection. The state-of-the-art operations include nonuser-defined parameter usage (e.g., model order [19,20,21]) through stabilization diagrams [22] and hierarchical clustering [11,23,24] within short-time fragments and eventually, modal anomaly detection in a continuing time frame. The proposed series of techniques are applicable for real-time identification with a competent acquisition framework; however, they are only presented with retrospective analyses in this paper.

Motivated by the state-of-the-art advances in automated modal analysis and anomaly-driven SHM, this study proposes a simultaneous anomaly detection procedure based on modal parameters using short-length vibration data collected from large-scale damaged and undamaged bridge cases. The proposed procedure requires no human intervention during the operation process. The effectiveness and limitations of this methodology are demonstrated in two large-scale bridge testbeds. The first example is Bridge 1, tested in the University of Nevada, Reno laboratories, and subjected to progressive damage resulting from several shaking table tests. The second example, Bridge 2, includes field tests of a pedestrian link bridge at Columbia University Morningside Campus, connecting two multistory buildings. Multi-input multioutput acceleration readings collected from Bridge 1 experiment comprise white noise and seismic excitations, whereas Bridge 2 is subjected to ambient vibration, where both cases are adopted for the anomaly detection procedure.

The rest of the paper is structured as follows. Section 2 introduces the methodologies describing system identification, stabilization diagram, clustering, and anomaly detection in a short-time setting, as well as the testbed bridges. Section 3 presents the implementation of the proposed framework with results and discussions through the testbed bridges, one damaged and another undamaged. Finally, Section 4 draws the conclusions obtained from the experimental and field findings with an emphasis on modal anomalies in individual and combined settings and threshold effects.

## 2. Materials and Methods

In this section, the methodological details and testbed information are expressed. The methodology starts with the linear time-invariant system identification, the SRIM method [21], and modal analysis supported by stabilization diagrams, and a clustering technique for short-time data segments. Afterwards, anomaly detection techniques and information retrieval metrics to quantify methodology performance are discussed. Eventually, two bridge structures, one experimental and another operational case are introduced as testbeds.

### 2.1. System Identification

#### 2.1.1. State-Space Model

A deterministic linear time-invariant system can be described by a state-space model as follows:

(1) $$x_{k+1}=Ax_{k}+Bu_{k}$$

(2) $$y_{k}=Cx_{k}+Du_{k}$$

where ***x_k_*** is an *n* × 1 state vector at time index *k* (*k* = 1:*l*), ***u****_k_* is an *r* × 1 input vector (*r* inputs), and ***y****_k_* is an *m* × 1 output vector (*m* sensors). System matrices **A**, **B**, **C**, and **D** are *n* × *n* system matrix, *n* × *r* input matrix, *m* × *n* output matrix, and *m* × *r* direct influence matrix, respectively.

A new realization algorithm SRIM using data correlation concept of shifted input and output [21] is introduced to identify system matrices directly from data. The input-state-output relation in Equations (1) and (2) can be performed for each time index *k* (*k* = 0, 1, 2, …, *l*) in a matrix form as follows:

(3) $$Y_{k}=OX_{k}+TU_{k},$$

where **O** $={\left[C;CA;CA^{2};\ldots ;CA^{i-1}\right]}^{im\times n}$ is an extended observability matrix (*i* > *n*) and **T** is a Toeplitz matrix,

(4) $$T={\left[\begin{array}{ccc} D & 0 & \begin{array}{ccc} 0 & \cdots & 0 \end{array}\\ CB & D & \begin{array}{ccc} 0 & \cdots & 0 \end{array}\\ \begin{array}{c} CAB\\ \begin{array}{c} \vdots \\ CA^{i-2}B \end{array} \end{array} & \begin{array}{c} CB\\ \begin{array}{c} \vdots \\ CA^{i-3}B \end{array} \end{array} & \begin{array}{ccc} \begin{array}{c} D\\ \begin{array}{c} \vdots \\ CA^{i-4}B \end{array} \end{array} & \begin{array}{c} \cdots \\ \begin{array}{c} \vdots \\ \cdots \end{array} \end{array} & \begin{array}{c} 0\\ \begin{array}{c} \vdots \\ D \end{array} \end{array} \end{array} \end{array}\right]}^{im\times ir},\;U_{i}\left(k\right)={\left[\begin{array}{ccc} u_{k} & u_{k+1} & \begin{array}{ccc} u_{k+2} & \cdots & u_{k+j-1} \end{array}\\ u_{k+1} & u_{k+2} & \begin{array}{ccc} u_{k+3} & \cdots & u_{k+j} \end{array}\\ \begin{array}{c} \vdots \\ u_{k+i-1} \end{array} & \begin{array}{c} \vdots \\ u_{k+i} \end{array} & \begin{array}{ccc} \begin{array}{c} \vdots \\ u_{k+i+1} \end{array} & \begin{array}{c} \vdots \\ \cdots \end{array} & \begin{array}{c} \vdots \\ u_{k+i+j-2} \end{array} \end{array} \end{array}\right]}^{ir\times j},\;Y_{i}\left(k\right)={\left[\begin{array}{ccc} y_{k} & y_{k+1} & \begin{array}{ccc} y_{k+2} & \cdots & y_{k+j-1} \end{array}\\ y_{k+1} & y_{k+2} & \begin{array}{ccc} y_{k+3} & \cdots & y_{k+j} \end{array}\\ \begin{array}{c} \vdots \\ y_{k+i-1} \end{array} & \begin{array}{c} \vdots \\ y_{k+i} \end{array} & \begin{array}{ccc} \begin{array}{c} \vdots \\ y_{k+i+1} \end{array} & \begin{array}{c} \vdots \\ \cdots \end{array} & \begin{array}{c} \vdots \\ y_{k+i+j-2} \end{array} \end{array} \end{array}\right]}^{im\times j},$$

with *i* integer defined by users (*i* > *n*/*m* + 1), *n* is the desired system order. The state matrix $X_{k}={\left[x_{k},x_{k+1},\ldots ,x_{k+j-1}\right]}^{n\times j}$ with integer *j* is chosen such that *j* = *l* − *k* − *i* + 2 covering the data length *l*.

#### 2.1.2. State-Space Model Realization

To determine system matrices **A**, **C** from **O** and **B**, and **D** from **T**, following autocorrelation and cross-correlation matrices are used to eliminate either **T** or **O** from Equation (3)

**R_yy_** = (1/*j*)**Y***_k_***Y***_k_*^T^, **R_uu_** = (1/*j*)**U***_k_***U***_k_*^T^, **R_xx_** = (1/*j*)**X***_k_***X***_k_*^T^ (5)

**R_yu_** = (1/*j*)**Y***_k_***U***_k_*^T^, **R_yx_** = (1/*j*)**Y***_k_***X***_k_*^T^, **R_xu_** = (1/*j*)**X***_k_***U***_k_*^T^ (6)

Post-multiplying both sides of Equation (3) by (1/*j*)**U***_i_*^T^, (1/*j*)**Y***_i_*^T^, and (1/*j*)**X***_i_*^T^ yields Equations (7)–(9), respectively

**R_yu_** = **OR_xu_** + **TR_uu_** (7)

**R_yy_** = **OR**^T^**_yx_** + **TR**^T^**_yu_** (8)

**R_yx_** = **OR_xx_** + **TR**^T^**_xu_** (9)

Substituting **T** = (**R_yu_** − **OR_xu_**)**R**^−1^**_uu_** (if **R_uu_** > 0) deduced from Equation (7) into Equations (8) and (9) returns

(10) $${\bar{R}}_{\mathrm{yy}}=O{\bar{R}}_{\mathrm{xx}}O^{T}$$

(11) $$\mathrm{where}\;{\bar{R}}_{\mathrm{yy}}=R_{\mathrm{yy}}-R_{\mathrm{yu}}R_{\mathrm{uu}}^{-1}R_{\mathrm{yu}}^{T},$$

(12) $${\bar{R}}_{\mathrm{xx}}=R_{\mathrm{xx}}-R_{\mathrm{xu}}R_{\mathrm{uu}}^{-1}R_{\mathrm{xu}}^{T}.$$

Then, taking singular value decomposition of the *im* × *im* matrix ${\bar{R}}_{\mathrm{yy}}$ yields

(13) $${\bar{R}}_{\mathrm{yy}}=\left[U_{1},U_{2}\right]\left[\begin{array}{cc} S & 0\\ 0 & 0 \end{array}\right]\left[\begin{array}{c} U_{1}^{T}\\ U_{2}^{T} \end{array}\right]=U_{1}SU_{1}^{T}.$$

Equations (10) and (13) imply **O** = **U**_1_. With **O** known, matrices **A** and **C** can be easily identified from the observability matrix $O={\left[C;CA;CA^{2};\ldots ;CA^{i-1}\right]}^{im\times n}$. To determine **B** and **D** from **T**, the matrix **O** should be eliminated from Equation (3). Postmultiplying Equation (3) by the *im* × *n* matrix **U**_1_^T^ and utilizing orthogonality property of **U**_1_ (**U**_1_ = **O**) and **U**_2_ produces

(14) $$T=R_{yu}R_{\mathrm{uu}}^{-1}$$

The Toeplitz matrix **T** in Equation (3) can be partitioned into parts to extract matrices **B** and **D**.

#### 2.1.3. Modal Parameter Identification from State-Space Matrices

The modal parameters as frequencies, damping ratios, and mode shapes are identified from the state-space matrices **A** and **C**. The system matrix **A** can be decomposed into eigenvectors and eigenvalues as follows:

(15) $$A=VSV^{-1}$$

with **S** (*n* × *n*) is a diagonal matrix containing eigenvalues *μ_i_* and **V** (*n* × *n*) is an eigenvector matrix. The eigenfrequency $f_{i}$, damping ratio $\xi_{i}$, and mode shape $\phi_{i}$ are then determined by

(16) $$f_{i}=ln\left(\mu_{i}\right)/dt,$$

(17) $$\xi_{i}=-\mu_{i}^{r}/\left|\mu_{i}\right|,$$

and

(18) $$\phi_{i}=CV,$$

with $\mu_{i}^{r}$ is the real part of a complex number and $\left|\mu_{i}\right|$ is a complex modulus.

The time-domain system identification procedure determining modal parameters depends on system order selection. Singular values as a function of system orders indicate the proper order where a sudden value drop is observed. However, in extreme noise cases, such a drop may not be prevalent, and further steps may need to be pursued, such as a stabilization diagram for accurate identification. The stabilization diagram reveals the repetitiveness of modal behavior at a series of different system orders, and stable poles can be distinguished based on the consistency over the domain.

### 2.2. Stabilization Diagram and Clustering

#### 2.2.1. Stabilization Diagram

Stabilization diagram is described by poles satisfying one or more criteria with different limits regarding frequency, damping ratio, and mode shape.

(19) $$\frac{f_{i+1}-f_{i}}{f_{i}}<lim_{f}\left(\%\right)$$

(20) $$\frac{\xi_{i+1}-\xi_{i}}{\xi_{i}}<lim_{\xi }\left(\%\right)$$

(21) $$MAC_{i,i+1}=\frac{{\left|\emptyset_{i}^{T}\emptyset_{i+1}\right|}^{2}}{\left(\emptyset_{i}^{T}\emptyset_{i}\right)\left(\emptyset_{i+1}^{T}\emptyset_{i+1}\right)}<lim_{MAC}\left(\%\right)$$

where *i* and *i* + 1 are consecutive system orders; *f* is frequency; *ξ* is damping ratio; ϕ is mode shape; (.)*^T^* is a complex conjugate transpose; $lim_{f}$, $lim_{\xi }$, and $lim_{MAC}$ are limits defined by users. The MAC measure ranges in [0,1] where MAC equal to zero indicates two different mode shapes, conversely MAC equal to 1 points out two identified modes to be the same mode. By default, this study uses the chosen limit measures associated with frequency, damping ratio, and MAC as 0.01, 0.05, and 0.05, respectively.

The stabilization diagram presents the frequency vs. system order relationship where modal parameters are estimated for different system orders. The population of identified modal parameters is categorized into four definitions of poles. They include poles stable in frequency satisfying Equation (19), poles stable in both frequency and damping ratios satisfying Equations (19) and (20), poles stable in frequency and mode shape satisfying Equations (19) and (21), and finally, stable poles satisfying all criteria in Equations (19)–(21). The stable poles considering all measures are typically used to identify modal parameters via visualized vertical lines from the stabilization diagram.

Visual modal identification from the stabilization diagram is suitable to apply for a few datasets. In cases of modal identification for many different datasets, the automation process needs to be developed. In the next section, the authors propose an automatic modal identification procedure for small datasets, where applied techniques in previous studies are also referred.

#### 2.2.2. Procedure of Identification Automation

The stabilization interpretation process can be compacted into two steps, including (1) diagram clearance and (2) clustering. The clearance step can be optional, depending on measured data quality and the identification algorithm. This step contributes to clearing nonphysical modes such as mathematical and noisy modes from the diagram. For that sake, different measures considering mode shape complexity, e.g., modal phase collinearity [25], mean phase deviation [26], contribution level/energy of modes to the total response, e.g., modal transfer norm [27], or modal energy level [28] can well enhance the clarity of the diagram. The next step is to recognize patterns in the diagram via clustering. In other words, clustering aims to automatically group modes with similar features into single clusters. In the literature, clustering techniques can be categorized into two groups, including hierarchical clustering [11,23,24] and nonhierarchical clustering or k-means clustering [29,30]. This step may require additional validation criteria, such as the object number in the recognized clusters, to select expected modes among physical modes.

The automated identification procedure in this paper applies the hierarchical clustering technique. The study follows the successful application of the hierarchical clustering algorithm for automatic modal identification of a long span arch bridge using high signal-to-noise observation data by Magalhaes [11]. In Magalhaes’s study, characteristics similarity of stable poles in the stabilization diagram is measured first using a Euclidean distance considering natural frequency and mode shape. The similar modes are then hierarchically sorted into separated clusters by calculating the shortest distance between any poles from one cluster to others. Finally, damping ratios are taken into consideration by eliminating outlier poles within each cluster, and the resulting clusters satisfy the minimum pole number defined by users.

There are two main difficulties in this study, which may require extra steps and changes in the context of automation. The first one is modal identification from small-sized datasets of real-time observation data may cause a shortage of stable poles in the stabilization diagram. Note that stable poles are modes satisfying all criteria in terms of frequency, damping ratio, and mode shape following Equations (19)–(21). The second one is noisy observation data from both experimental and operational modal analysis, which may confuse wanted clusters with others, are caused by noise and mathematical superiority. Because of these obstacles, the following steps for the automated identification process are implemented, and references are made to previous studies where is necessary.

The process starts with poles stable in only natural frequency and mode shape among four different sorts of poles in the stabilization diagram: 1—poles stable in frequency, 2—poles stable in frequency and damping ratio, 3—poles stable in frequency and mode shape, and 4—stable poles satisfying all criteria. Note that stable poles satisfying all the above-defined limits regarding frequency, mode shape, and damping ratio are not considered during the process due to their shortage possibly estimated from small datasets. However, the damping ratio associated with those poles is also considered in the final step through an outlier detection method.

Step 1: Clearing the stabilization diagram of poles stable in frequency and mode shape by dropping all poles with negative damping ratio and excluding spurious modes by Modal Phase Collinearity (MPC). It is noted that this step is optional depending on the collected data quality and the applied system identification method. The MPC is used to examine the purity of each mode via spatial consistency of phase angles, developed by Pappa et al. [25]. Suppose that $\phi_{r}$ and $\phi_{i}$ are real and imaginary parts of the *i*th identified mode, respectively, the variance and covariance of $\phi_{r}$ and $\phi_{i}$ can be calculated as follows:

(22) $$S_{rr}=\emptyset_{r}^{T}\emptyset_{r}$$

(23) $$S_{ii}=\emptyset_{i}^{T}\emptyset_{i}$$

(24) $$S_{ri}=\emptyset_{r}^{T}\emptyset_{i}$$

Letting $\eta =\frac{S_{rr}-S_{ii}}{2S_{ri}}$, the eigenvalues of the variance/covariance matrix can be computed as:

(25) $$\lambda_{1,2}=\frac{S_{rr}+S_{ii}}{2}\pm S_{ri}\sqrt{\eta^{2}+1}$$

The MPC for the *i*th mode is formulated as follows:

(26) $$MPC_{i}={\left(\frac{\lambda_{1}-\lambda_{2}}{\lambda_{1}+\lambda_{2}}\right)}^{2}\times \left(100\%\right)$$

The MPC value ranges from 0 to 1, where 0 represents spurious modes (modes with uncorrelated phase angles) and 1 indicates physical modes (modes with perfectly correlated phase angles). Some other works applied MPC [31] or combined MPC and mean phase deviation (MPD) [12,24] to eliminate spurious modes from the stabilization diagram.

Step 2: The hierarchical clustering algorithm is applied to recognize all modes with similar characteristics as a single mode [32,33]. The algorithm that starts with each system pole, herein, a pole stable in frequency and mode shape, is considered as a separate cluster. The process then sorts two closest poles into one single cluster by a distance measure or also called a cluster threshold. The threshold choice depends on users and is presented in later details. The process repeats one by one until the distance between the remaining clusters is larger than the user-defined threshold. The resulting clusters are chosen based on the required minimum pole number in each cluster. The threshold value selection and the minimum number of each cluster are often varying on different datasets. This is one of the disadvantages of the automatic identification process in real-time problems where modal parameters are identified from different small datasets. In this study, the difficulty is resolved by a tradeoff related to simplicity and computational cost between the cluster threshold choice and the required minimum number of each cluster. For this reason, the cluster threshold is fixed, and the other is changeable to recognize at least one cluster for each segment. More importantly, this compensation does not influence anomaly detection results as the ultimate purpose of the study.

Step 3: Removing outliers in terms of damping ratio within each recognized cluster in step 2. The median absolute deviation (MAD) [34] is used to cancel any outliers from each cluster *j*.

(27) $$MAD_{j}=\alpha \times Md\left(\left|x_{i}-Md\left(x_{i}\right)\right|\right),$$

with coefficient α=1.482, *Md* is median, and $x_{i}$ is pole stable in frequency and mode shape. This simple step can be considered as a validation step to avoid modes with extreme values of damping ratios, which do not represent the real structural behavior.

### 2.3. Anomaly Detection

Anomaly algorithm starts with an unlabeled dataset, also referred to as a reference dataset. The collected data is supposed to have a vast number of nonanomalous points, but there may be some anomalous points as well. Herein, the anomaly detection algorithm is explained with a dataset in *n* dimensions, {*x*^(1)^, *x*^(2)^ …, *x*^(*m*)^}, where $x^{\left(i\right)}\in {\mathbb{R}}^{n}$.

(i). Fit a Gaussian distribution to the data {*x_j_*^(1)^, *x_j_*^(2)^ …, *x_j_*^(*m*)^} for each *j*-th dimension feature $x_{j}$ and *j* = 1, …, *n*. The Gaussian distribution is given by
  (28) $$p\left(x_{j},\mu_{j},\sigma_{j}^{2}\right)=\frac{1}{\sqrt{2\pi \sigma_{j}^{2}}}e^{-\frac{{\left(x_{j}-\mu_{j}\right)}^{2}}{2\sigma_{j}^{2}}},$$
  where the mean is
  (29) $$\mu_{j}=\frac{1}{m}\sum_{i}^{m}x_{j}^{\left(i\right)}$$
  and variance is
  (30) $$\sigma_{j}=\frac{1}{m}\sum_{i}^{m}{\left(x_{j}^{\left(i\right)}-\mu_{j}\right)}^{2}$$
(ii). Calculate the probability of a new data point, *p*(*x*), in multiple dimensions based on the above estimated Gaussian distribution,
  (31) $$p\left(x\right)=\prod_{j=1}^{n}p\left(x_{j},\mu_{j,}\sigma_{j}^{2}\right)=\prod_{j=1}^{n}\frac{1}{\sqrt{2\pi \sigma_{j}^{2}}}e^{-\frac{{\left(x_{j}-\mu_{j}\right)}^{2}}{2\sigma_{j}^{2}}}$$
(iii). Compare *p*(*x*) with a threshold *ε*, if *p*(*x*) < *ε*, the data point *x* is an anomaly.

Given the above equations, combined univariate anomalies can be detected as an alternative to multivariate approaches. For the simplest case where *n* equals 1, the situation corresponds to univariate anomaly detection, referred to as Grubb’s test [35,36]. The threshold value *ε* thus defines deviation from the mean, *kσ*, where *k* is an integer (*k* = 1, 2, 3 …). The optimal threshold is then suggested to detect anomalies of the structures depending on considered damage levels.

**Figure 1:**
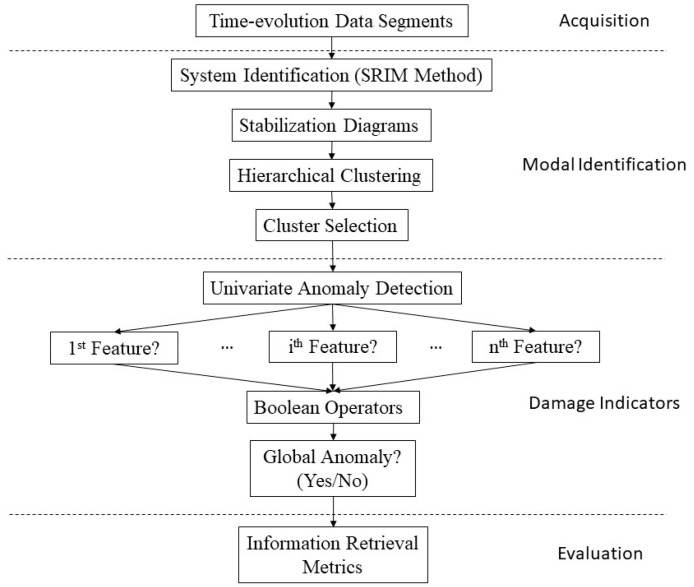
Automated modal parameter-based anomaly detection procedure.

In other words, features reflect individual modal parameter anomalies (e.g., *i*th modal frequency) where the thresholds are exceeded. Boolean operators (e.g., “and” and “or”) then combine these features for a resultant anomaly depending on the operator’s architecture. Anomaly detection results can be classified into four different types, including true positive (TP), true negative (TN), false positive (FP), and false negative (FN). The “true” and “false” terms indicate right and wrong of predicted anomalies, whereas “positive” and “negative” terms represent abnormal and normal structural states, respectively. To bring all the above possible outcomes together, eight information retrieval metrics, as shown in Table 1, are used to evaluate the reliability of detected anomalies. The combined automated procedure of modal identification and anomaly detection is summarized in Figure 1.

### 2.4. Testbeds

In this study, two large-scale bridge tests are referred to as testbeds. One of them corresponds to a reinforced concrete bridge subjected to incremental damage, whereas another one is an undamaged pedestrian link bridge. The details of testbeds are explained in the next subsections.

#### 2.4.1. Structure 1: RC Bridge Subjected to Earthquakes

**Figure 2:**
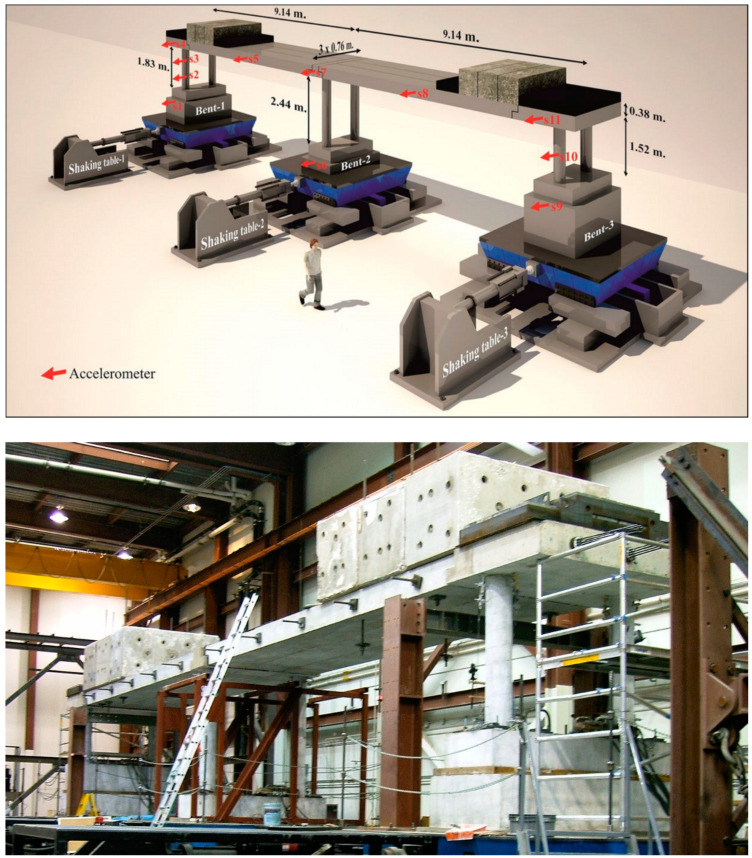
Experimental setup and sensor layout of the Bridge 1 test, conducted at University of Nevada, Reno [39].

The simultaneous anomaly detection framework is firstly applied on a large-scale shake table test of a bridge experiment conducted at the University of Nevada, Reno. Figure 2 shows the testing specimen, a 20-m long two-span reinforced concrete bridge subjected to incremental seismic damage. Additional masses weighing a total of 1190 kN are added on top of the outer bents to represent the adjacent spans of a typical bridge. The deck rests on three bents, each composed of two columns fixed to the individual bases subjected to shaking. The structure is exposed to three sequential earthquake events and low-amplitude white noise excitations between each earthquake. Vibration excited from a shaking table separated under each bent is assumed to synchronize to the same target time history. Eleven FBA-11 Kinemetrics accelerometers are placed on the shaking tables, bent columns, and bridge deck to measure input ground motion and structural response in the excitation direction. These tests are conducted with a sampling rate of 200 Hz. The detailed report and condensed review of the testing procedure can be found in [37,38]. To avoid direct current components, bias, and aliasing effects, the vibration signals are band-pass filtered with lower and upper cutoff frequencies of 0.5 and 49 Hz, respectively.

**Figure 3:**
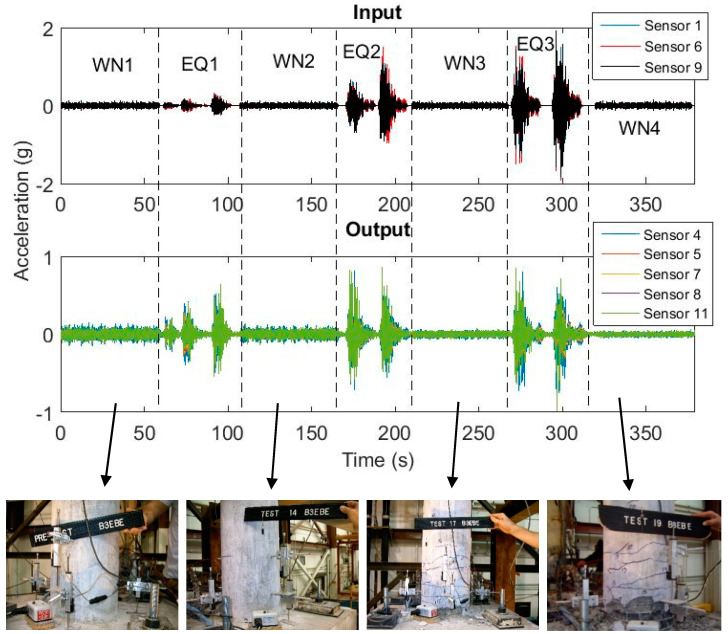
Acceleration time history of input/output measurements of Bridge 1 and accompanying damage photographs from Bent 3 bottom section [39] (WN: white noise; EQ: earthquake).

Figure 3 shows the input ground motion measured from Sensor 1, 6, and 9 placed on the shaking tables and output response time histories from Sensor 4, 5, 7, 8, and 11 positioned on the bridge deck, including four white noise and three consecutive earthquakes. Ground motion intensities and corresponding damage levels are summarized in Table 2. The acceleration response is divided into overlapping short time windows and used for simultaneous system identification.

#### 2.4.2. Structure 2: Steel Pedestrian Link Bridge

**Figure 4:**
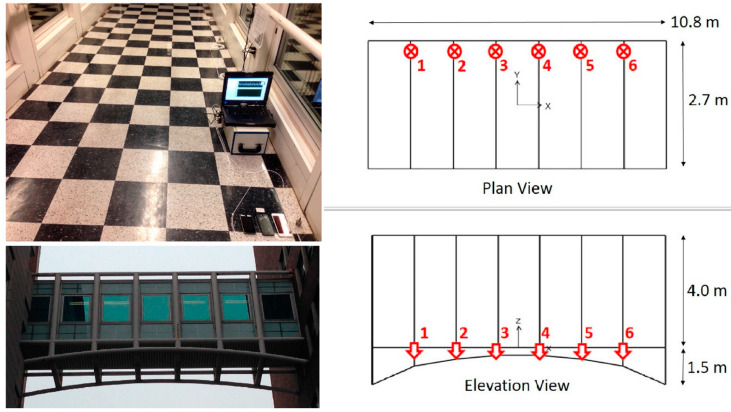
Inner, outer views, and sketches of Bridge 2, connecting Mudd-Schapiro Buildings.

**Figure 5:**
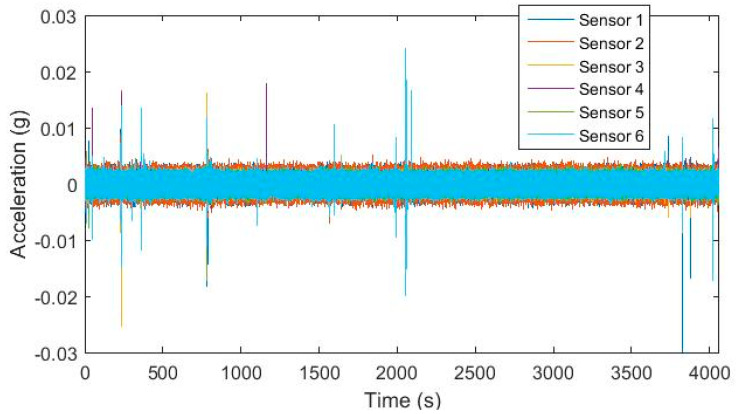
Acceleration time history at Sensors 1–6 of Bridge 2.

The second structure considered in this study is a steel pedestrian link bridge located at the Morningside Campus of Columbia University, NY. The bridge is evenly instrumented with six piezoelectric (393B04 model, PCB Piezotronics, New York, NY, USA) accelerometers oriented in the vertical direction on one side of the bridge to acquire low-amplitude response data for system identification. In other words, the structure has no damaging experience throughout the tests. More than 1-h acceleration data is obtained at nighttime to minimize pedestrian-induced vibration, and measurements are collected under ambient vibration. The data is sampled at 100 Hz, and band-pass is filtered with cutoff frequencies of 0.5 and 49 Hz. Figure 4 and Figure 5 demonstrate the bridge instrumentation and six-channel acceleration response under ambient vibration, respectively.

## 3. Results and Discussions

This section presents the implementation of expressed automated modal analysis, anomaly detection, and performance metrics techniques on the two bridge cases introduced in the previous section. First, simultaneous modal analysis results are presented, including system identification, stabilization diagram, and clustering. Afterwards, instantaneously identified modal parameters are used for anomaly detection, and findings are evaluated according to information retrieval criteria.

### 3.1. Automated Extraction of Modal Parameters

#### 3.1.1. Automated Modal Parameters Extraction from Bridge 1

Input and output response acceleration data collected from the Bridge 1 during all the tests are divided into small 10-s segments with 75% overlapped time between two segments. Modal parameters, including natural frequencies, modal damping ratios, and mode shapes, are identified for each of the total 148 segments by the SRIM method. The modal parameters are assumed to be unchanged over such short-time data segments, even though nonlinear responses could occur during large excitations. The stabilization diagrams presenting the relationship between natural frequency vs. system orders are generated for all segments. The modal parameters can then be selected by visualizing vertical lines showing the stability of natural frequencies against different system orders. As a result, the modal parameters selection is independent of the system orders, leading to more robust results.

**Figure 6:**
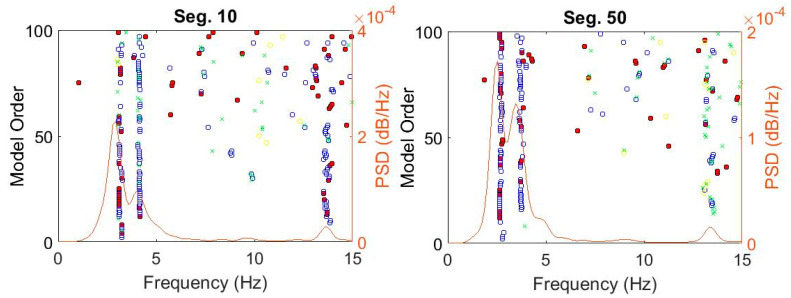

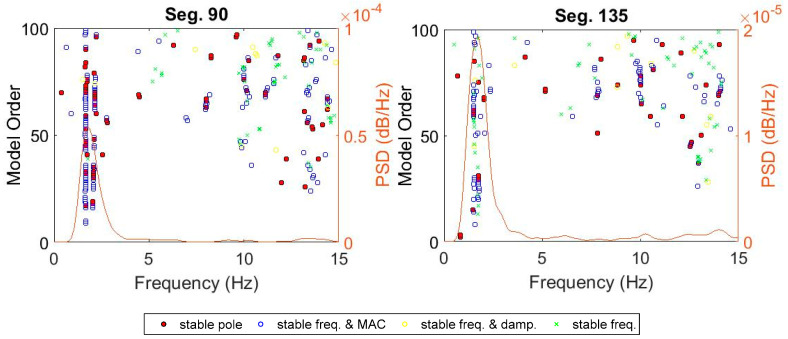
Stabilization diagrams for data segment number 10, 50, 90, and 135 of Bridge 1.

Figure 6 shows stabilization diagrams of four segments 10, 50, 90, and 135 representing different structural states, consisting of intact, low, medium, and high damage levels, respectively. The diagrams display four kinds of poles, including poles stable in only frequency, frequency vs. damping ratio, frequency vs. mode shape, and combined all three parameter criteria, as defined in Section 2.2.1. Due to the scattered nature of damping ratios compared with the others, poles associated with frequency and MAC are more dominant, as depicted in Figure 6. Furthermore, the number of estimated poles is also restricted due to the incompleteness to capture all significant modes from short-time data segments. For this reason, the poles stable in frequency and MAC are favorably adopted for selecting critical modes through recognizing vertical lines in the diagram. The selection process of these vertical lines can be performed automatically via the clustering technique.

The hierarchical clustering is applied for only poles stable in frequency and MAC. The automation identification procedure for Bridge 1 is performed in 3 steps following Section 2.2.2. Following that procedure, all the diagrams generated from all 148 segments are cleared by eliminating negative damping ratios without using MPC since the data collected from the laboratory experiment is supposed to be low noise measurement. After purifying the diagrams, the hierarchical clustering is applied. The maximum distance between two poles or the cluster threshold is fixed at 0.5 for the automation of the algorithm implementation.

**Figure 7:**
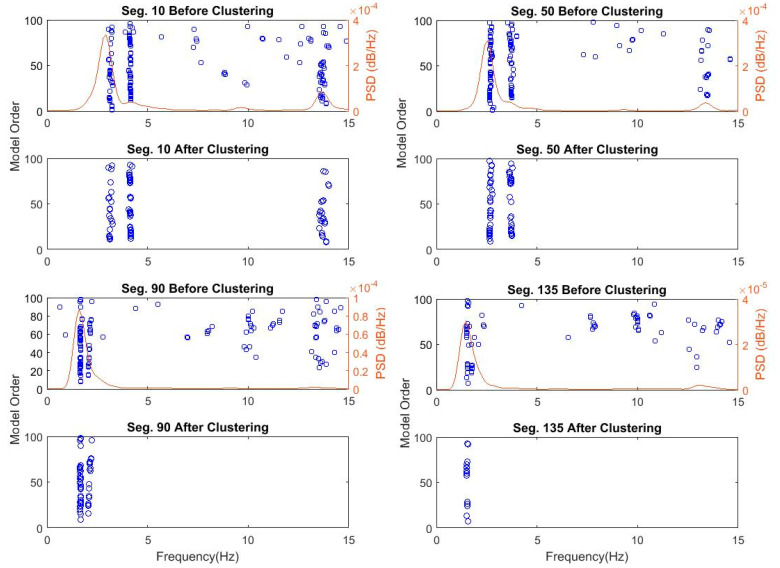
Figures showing poles stable in frequency and mode shapes only and identified clusters for segments 10, 50, 90, and 135 of Bridge 1.

**Figure 8:**
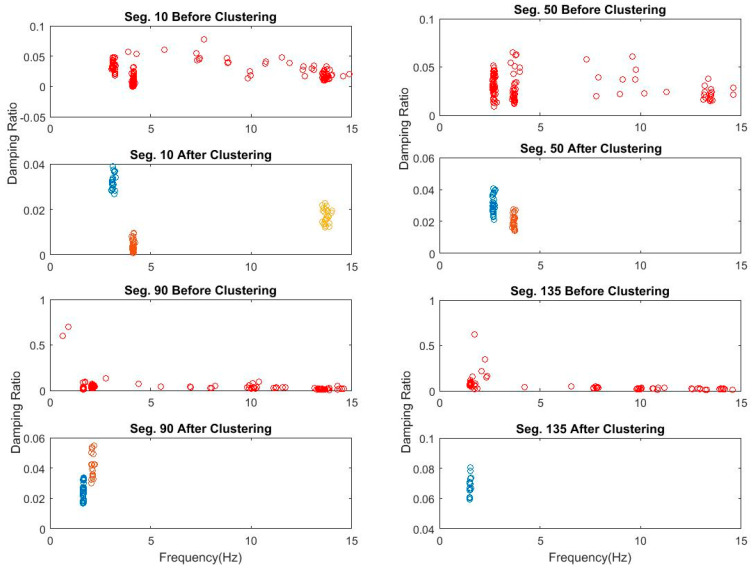
Bridge 1 damping ratio and frequency diagram of poles stable in frequency and mode shape of segments 10, 50, 90, and 135 before clustering and after clustering.

In contrast, the number of objects in each cluster is set changeable from a threshold value of 5 to lower values until at least one cluster is recognized. Figure 7 shows the recognized clusters for 4 data segments 10, 50, 90, and 135 processed through Steps 1 and 2. Finally, identified clusters are validated by removing outliers regarding damping ratios using the MAD measure, as shown in Figure 8. The figure shows the successful removal of nonphysical poles associated with extreme damping ratios that are larger than 10%.

**Figure 9:**
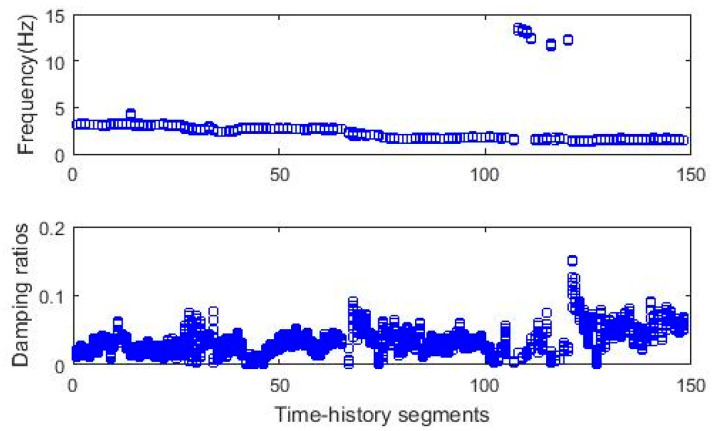
Time-dependent modal identification results (fundamental frequency, Bridge 1).

Figure 9 shows natural frequencies and damping ratios collected from the first recognized cluster for all 148 segments. As referred in [40,41], the first three modes of the undamaged structure are approximately in ranges of 2.5–3.2, 3.5–4.2, and 13.5–14.2 Hz. The selected first cluster, as shown in Figure 9, indicates the first mode, with some exemptions, e.g., the second mode captured for segment No. 14 during the healthy condition and the third mode for segments No. 108–111, 116, and 120 during the third damaging event. The first clusters showing damping ratios are more scattered than the ones regarding frequency. These clusters are adopted for the anomaly detection in Section 3.2.1.

#### 3.1.2. Automated Modal Parameters Extraction from Bridge 2

**Figure 10:**
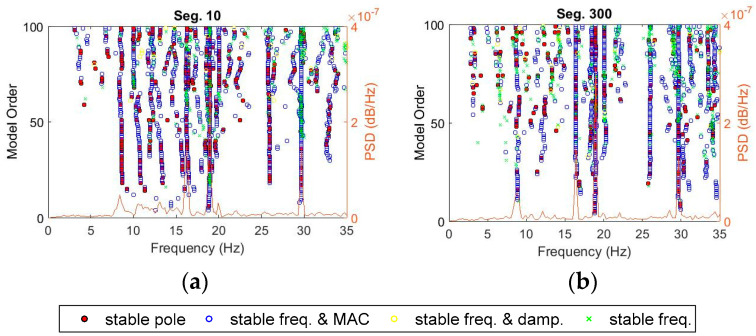
Stabilization diagram of time-history from Bridge 2: (**a**) seg. 10 and (**b**) seg. 300.

More than 1-h ambient response acceleration data collected from six smartphone sensors are divided into 20-s segments with 50% overlapped time between segments. The measured data is supposed to have a high signal-to-noise ratio since they are collected during operational ambient vibration. Through the SRIM identification method, natural frequencies, damping ratios, and mode shapes are identified for each of the total 405 data segments. Similar to the automated parameters extraction procedure for Bridge 1, the stabilization diagrams were generated from all segments of Bridge 2, and then the hierarchical clustering is applied to choose clusters automatically. Figure 10 shows the stabilization diagrams for segments No. 10 and 300. The diagrams are unclear due to the noisy measured data, thus, they need purification before clustering.

**Figure 11:**
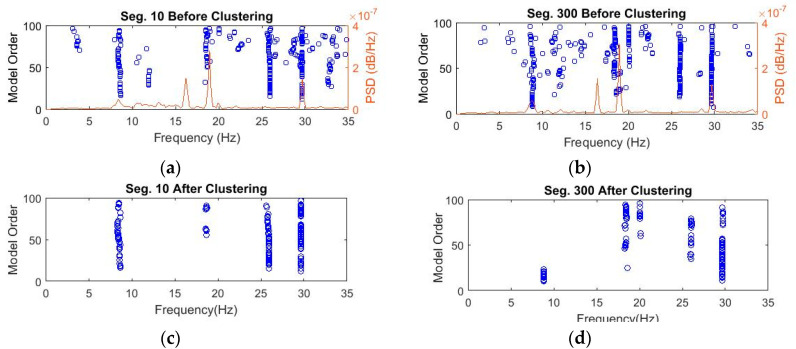
Bridge 2 (**a**,**b**) stable poles in frequency and mode shapes showing real modes for segments 10 and 300, and (**c**,**d**) resulting clusters.

**Figure 12:**
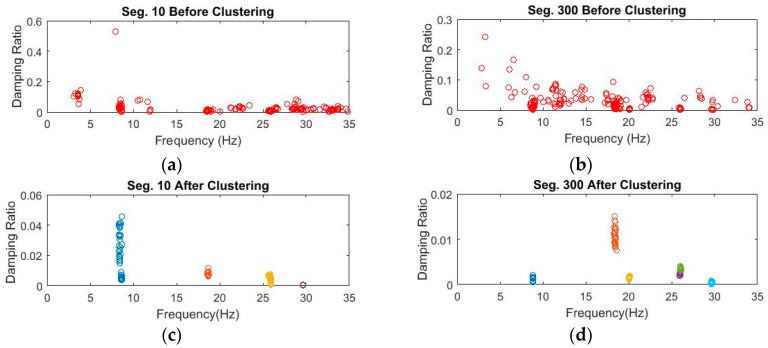
Bridge 2 damping ratio and frequency diagram of stable poles in frequency and mode shape for (**a**,**b**) before clustering, and (**c**,**d**) after clustering.

**Figure 13:**
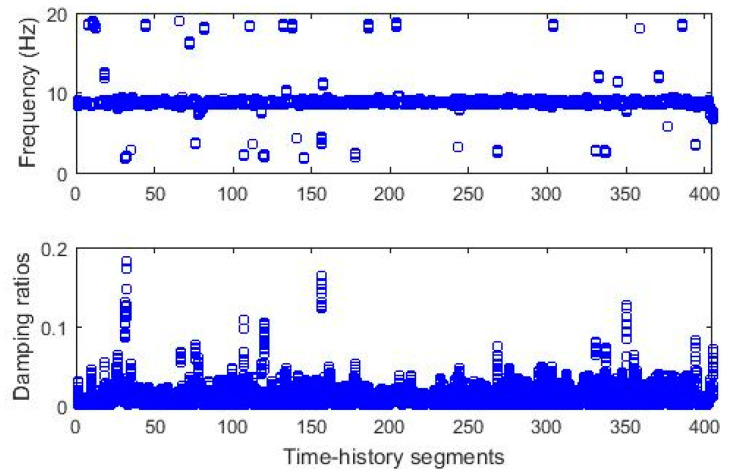
Time-dependent modal identification results (fundamental frequency, Bridge 2).

The clustering process follows three steps, including diagram clearance, clustering, and outlier removal, as presented in Section 2.2.2. Figure 11a,b shows the diagrams with poles stable in frequency and mode shape after removing nonphysical modes. The clearance step removes negative damping ratios and spurious poles using the MPC measure. Figure 11c,d displays the recognized clusters with a constant threshold value of 0.5. The last clusters after the outlier removal of extreme damping ratios are illustrated in Figure 12. The relationship between poles collected from the first clusters and segments is shown in Figure 13. The figure shows quite stable evolution via time, except for outliers since the modal identification for different data segments can result in different structural modes. Based on the identification results from the previous work [42], the first, second, and third modes are identified approximately as 8.4–8.6, 18.5–19.5, and 29.5–30.5 Hz, respectively. Figure 13 illustrates that the first cluster is corresponding to the first mode in general. These first clusters are used for anomaly detection in Section 3.2.2.

### 3.2. Identification of Modal Anomalies

In this subsection, simultaneous anomaly identification findings are presented with and without the combination of univariate modal features through simple Boolean operators. The presentation of findings from the two testbeds differs in the way input motion damage exposures take place.

For both bridges, different anomaly coefficients are expressed, and the initial time instances are considered to construct the original modal parameter distribution. The following instances producing new simultaneous modal parameters, later on, are examined according to the original distribution. In terms of modal features, identified frequency and damping ratio features in the previous sections are used.

For further explanation, Bridge 1 is subjected to incremental damage through a series of earthquake incidents which expectedly influences the anomaly detector positively in the latter instantaneous identification scenarios. On the other hand, Bridge 2 is exposed to no catastrophic event, which returns negative results from the anomaly detector. However, both cases are subjected to a compromise, depending on the quantity of the outlier coefficient.

#### 3.2.1. Bridge 1 with Sequentially Damaged States

Bridge 1, which experiences three sequentially incremental deteriorating events, considers three different referencing scenarios. The first scenario considers the pre-all-event case, where the original modal feature population is extracted from the stable identification values of the first 26 time instances. The second scenario disregards the first occurring low-amplitude earthquake, which corresponds to the top 69 time instances. The final scenario only initiates before the large-intensity earthquake, which occurs following the 109th time instant. Depending on the scenario and the original modal feature population, the anomaly detector returns different binary findings.

**Figure 14:**
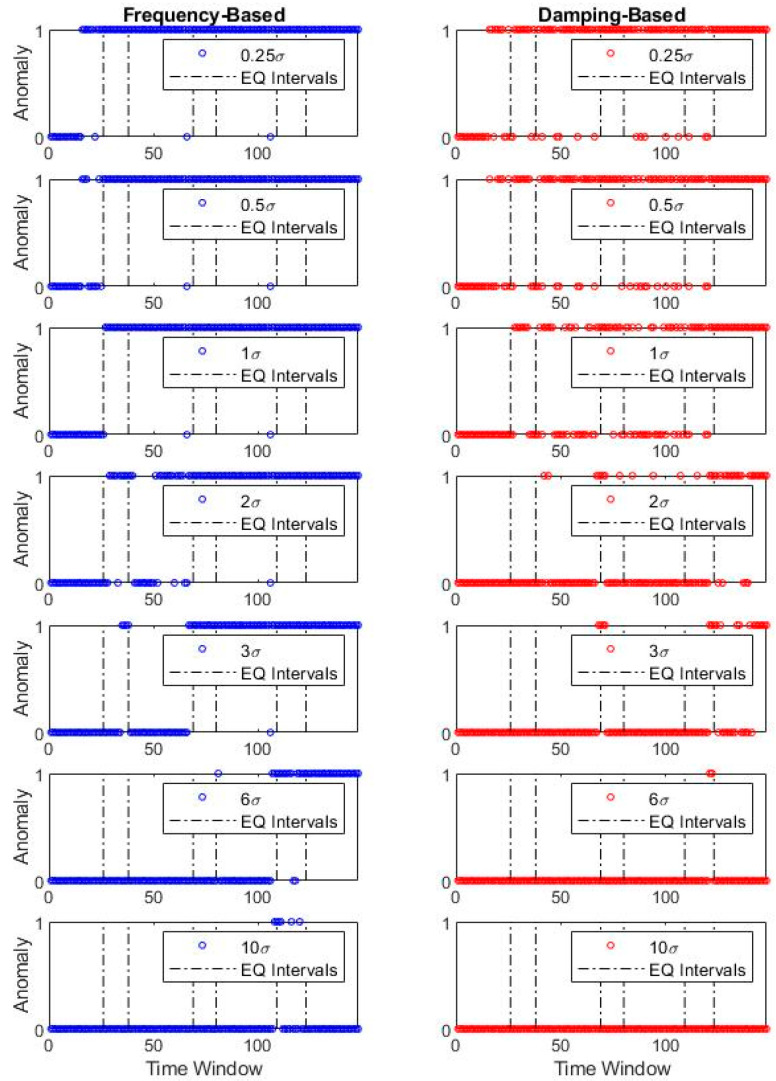
Bridge 1 simultaneous anomaly results for different σ coefficients (individual).

Figure 14 plots the anomaly values corresponding to the case, based on the original dataset before the first earthquake. The anomaly results depict that the σ coefficient serves as the sensitivity level determiner and influences the output accordingly. For low coefficient values (e.g., 0.25σ), the system has a low tolerance to modal parameter changes, whereas high coefficient values (e.g., 10σ) are insensitive to even significant damage. The remaining σ values denote the transition between ultimate sensitivity and ultimate tolerance extremes.

For case 1, where the original dataset stems from pre-earthquake-1, the optimal value can be qualitatively observed at σ equal to 1 for frequency-based detection. For a damping-based approach, the detector is incapable of drawing a strict line between the damaged and undamaged cases and thus presents numerous instances with false anomaly representation. When pre-earthquake-2 and pre-earthquake-3 cases are under consideration, frequency-based optimal σ moves to higher values such as 3 and 6, respectively. Similar to the first case, damping-based anomaly detection is less stable and involves smoother transitions between the damaged and undamaged states.

**Figure 15:**
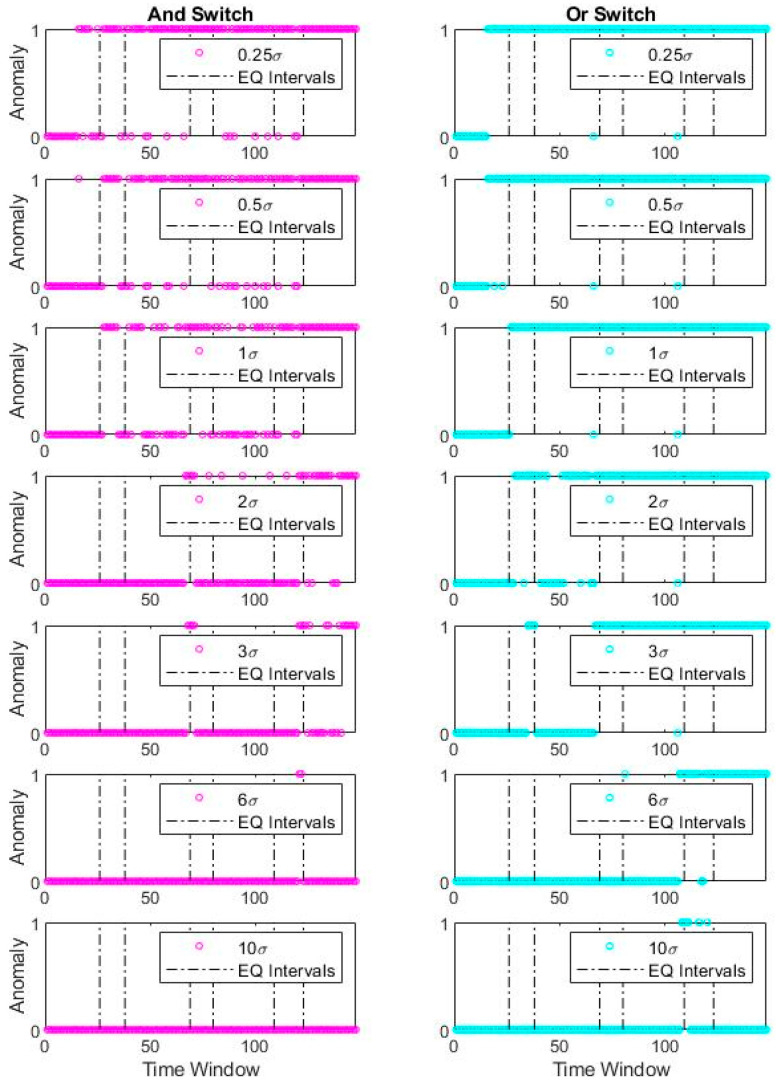
Bridge 1 simultaneous anomaly results for different σ coefficients (Boolean combined).

Said differently, it can be observed that frequency identifiers work relatively robust in contrast with the damping ratio, although the damping ratio also presents an unsharp transition at the damage instants. An incremental step to exercise is collocated usage of both identification results and see if the detection quality can improve. For this exercise, two simplistic merge scenarios are presented using “and” and “or” Boolean operators. For further explanation, “and” operator corresponds to the anomaly equal to 1 for the frequency-based and damping-based detectors. In contrast, only a single anomaly value equal to 1 is sufficient to activate the “or” operator. Thus, the proposed scheme studies a primitive combination of the univariate anomalies as a minimalist alternative to multivariate anomaly detection. Figure 15 shows the combined detection results of the two fundamental Boolean operators.

Interestingly, the “and” operator is almost identical to the damping-based univariate detector, whereas “or” operator resembles the frequency-based univariate detector. In other words, Boolean operator has an insignificant effect on the detectors, likely due to the dependence of the univariate detectors. Therefore, consideration of the joint distribution of damping ratio and frequency identification results might be essential to improve the conjunct consideration of parameters through a multivariate approach. Multivariate anomaly detection is expected to possess difficulties due to the different distribution features of the identified parameters (e.g., distribution types, skewness, and differences in uncertainty levels); however, it is promising if the statistical strategy is well posed.

**Figure 16:**
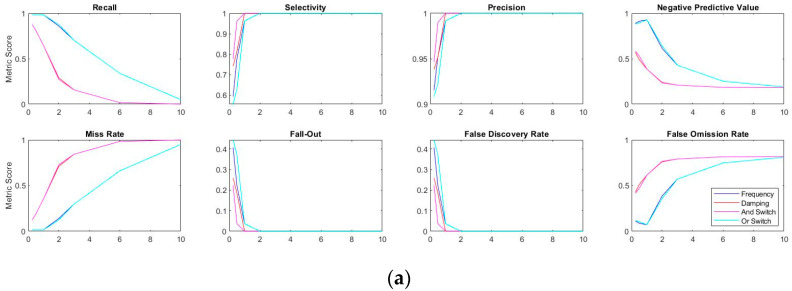

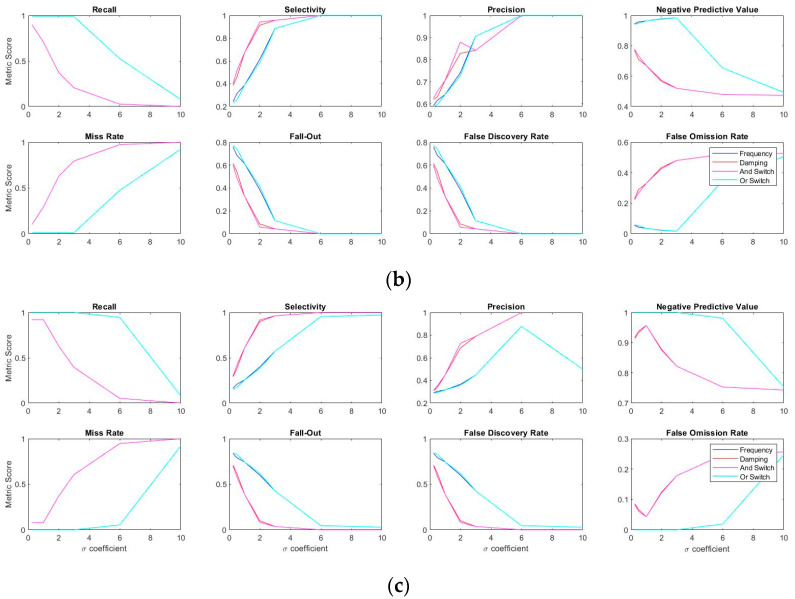
Information retrieval metrics as functions of σ under (**a**) first, (**b**) second, and (**c**) third quake cases.

For quantification of anomaly detection accuracies, several information retrieval metrics are taken into consideration. These include favored conditions such as recall, selectivity, precision, and negative predictive value, as well as undesirable metrics such as miss rate, fall-out, false discovery rate, and false omission rate. Expressed in the previous section, there are tradeoffs among information retrieval metrics (e.g., recall/negative predictive value performance decreases, whereas selectivity/precision increases with higher σ coefficients). Figure 16 shows the information metric scores as a function of σ coefficients under three different scenarios: pre-earthquake-1, pre-earthquake-2, and pre-earthquake-3 as the original modal feature population.

**Figure 17:**
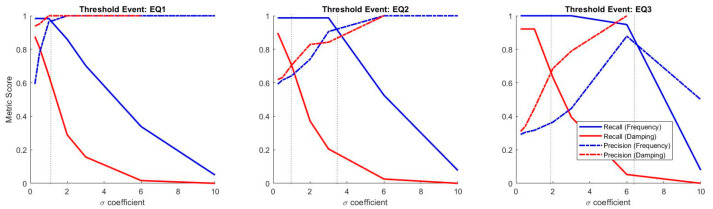
Bridge 1 intersecting information retrieval features of precision and recall metrics under first, second, and third earthquake scenarios.

According to the figure, both incremental and decremental plots show a leftwards shift from the first to the last earthquake scenario. In other words, considering the optimal σ coefficient where incremental metric meets decrement, the best result depends on the definition of an anomaly in terms of damage. In general, a more sensitive detector is needed for a high recall/negative predictive value score, which in turn, results in low selectivity/precision score. Taking the low-intensity first-earthquake into consideration, frequency-based recall and precision meet at the coefficient value around 1σ, whereas such intersection values are observed around 3.5σ and beyond 6σ for the medium-intensity second-earthquake and high-intensity third-earthquake, respectively. Such intersection values significantly reduce the damping-based anomaly detection, presented in Figure 17 for the readers’ convenience.

#### 3.2.2. Bridge 2 with No Damage Exposure

**Figure 18:**
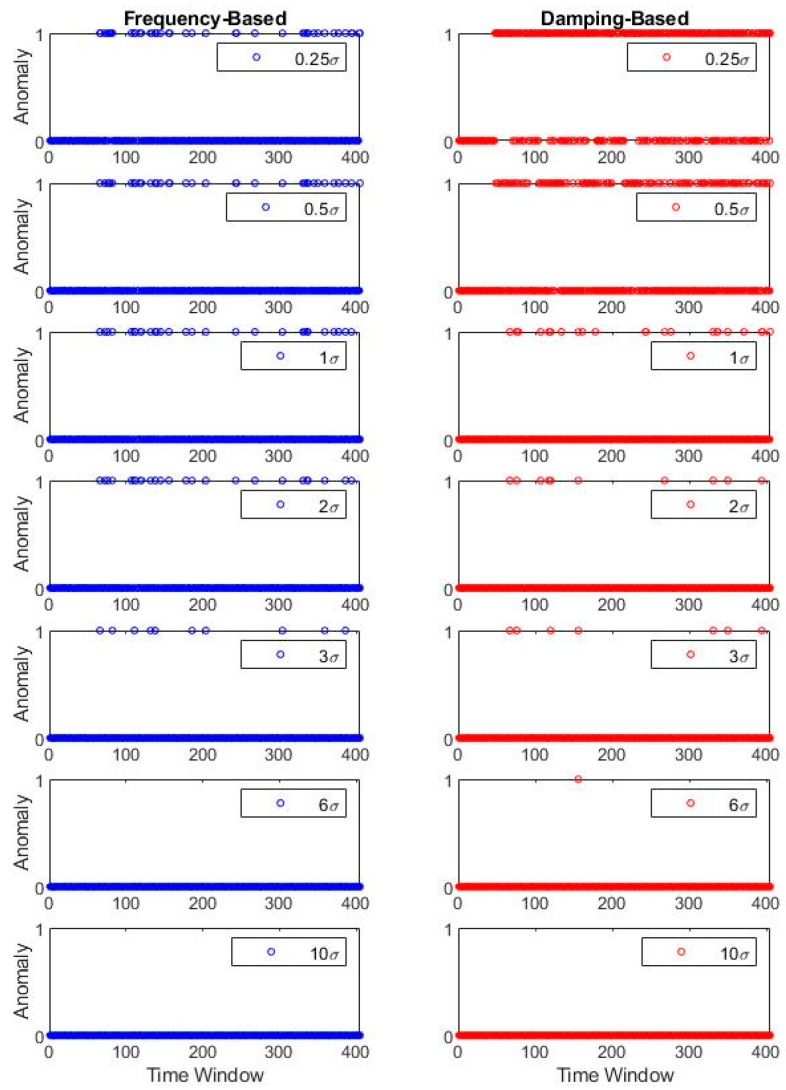
Bridge 2 simultaneous anomaly results for different σ coefficients (individual).

**Figure 19:**
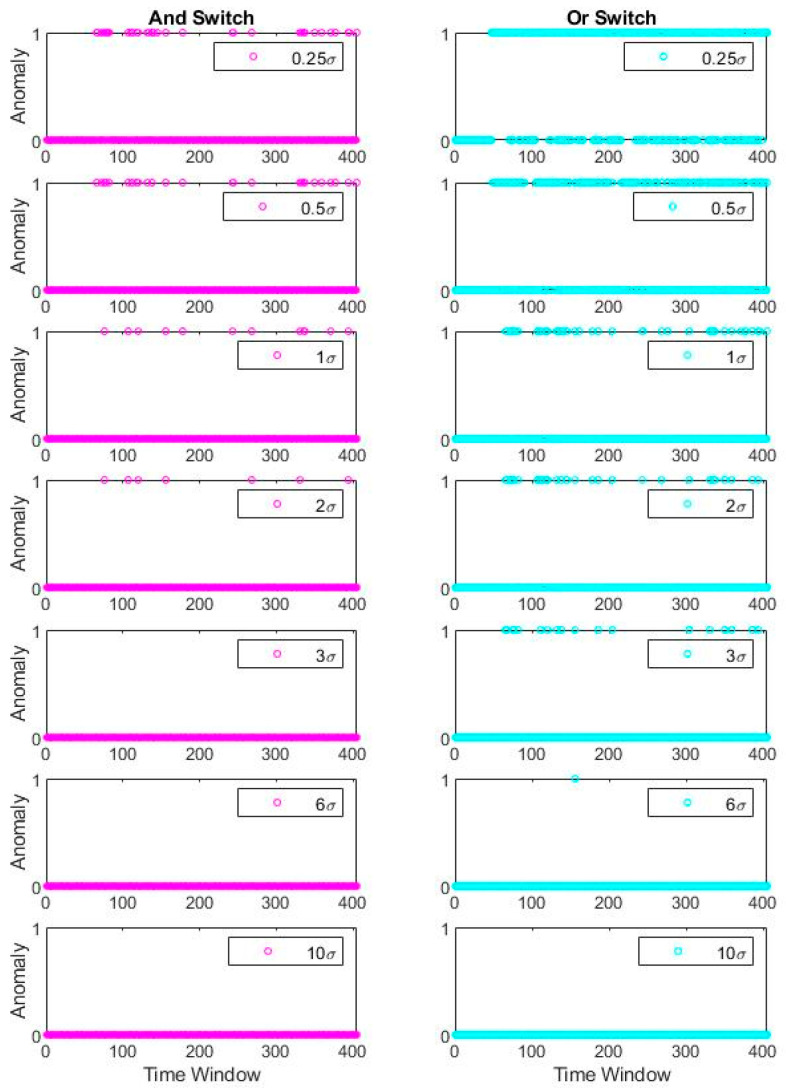
Bridge 2 simultaneous anomaly results for different σ coefficients (Boolean combined).

For reflecting a different aspect of the anomaly detection problem as a simultaneous SHM component, Bridge 2 with no damage is investigated. In real-life SHM scenarios, upon the instrumentation of a bridge, the original modal feature populations are restrained to healthy bridge data; therefore, the tradeoff between the incremental and decremental information retrieval metrics is excluded. The original population, as well as cross-check values, refers to negative anomalies that disables the optimal sigma coefficient balance; to specify, true positives are yet to exist. When there is a lack of true positives in the cross-checked dataset, specific information metrics are influenced in the nominator and denominator level. Restrictions exist due to healthy-only data. Figure 18 and Figure 19 plot the anomaly detection outputs of individual and Boolean-merged features, respectively.

**Figure 20:**
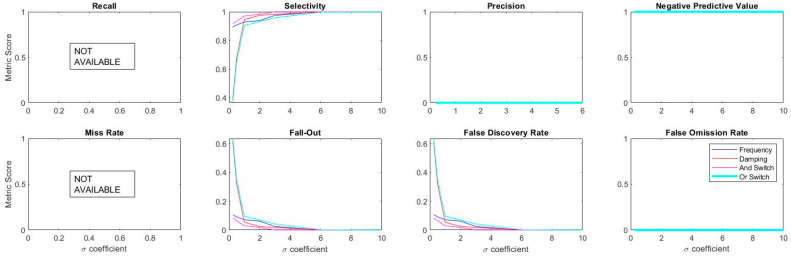
Information retrieval metrics as functions of σ.

The figures demonstrate that observing the performance of the monitoring system becomes open-ended since the sigma coefficients are higher, in other words, the less sensitive the detectors become, the better identification of negative anomaly. It possesses an identification weakness since the metrics such as recall and negative predictive value are likely to reduce as the sigma coefficient increases. Figure 20 quantifies the information retrieval metrics and presents the lack of tradeoff between different information retrieval metrics, some of which are unavailable (e.g., recall and miss rate). At the same time, some of them are redundant (e.g., precision, negative predictive value, and false omission rate) without the presence of positive anomaly values. However, the situation is a natural SHM problem, which initiates the anomaly detector without damage data. It should be noted that synthetic damaged data can be simulated, which again relies on modeling expertise and can be nontrivial to embed into data-driven techniques. Future studies need to seek solutions to cross-checking the dilemma to evaluate the desired sensitivity of modal anomaly detection.

It should be expressed that environmental effects can also trigger the anomalies depending on the threshold, which are excluded in this study. However, the literature has robust approaches incorporating this issue. A similar discussion on environmental effects is provided in [43].

## 4. Conclusions

In this paper, simultaneous anomaly detection with multimodal parameters is applied to two bridge case studies, one damaged and another undamaged throughout the tests. Bridge 1 corresponds to a large-scale reinforced concrete laboratory specimen subjected to consecutive and incremental seismic damage, whereas Bridge 2 remains intact throughout the acceleration response acquisition process. The acquired data are used for system and modal identification under overlapping short-duration time windows mimicking a streaming scenario. Each data subset, representing an instance, was processed through the SRIM method, then, stabilization diagram is developed, stable modal findings related to frequency and mode shape are clustered, and outliers regarding damping ratio are removed. Afterwards, anomaly detection is applied to the sequential instances taking the group of initial subsets as the reference distribution. Two modal parameters are pursued as anomaly identifiers, and simplistic Boolean combination approaches are presented for integrated usage of frequency and damping ratio features. The anomaly detection method is conducted under different deviation coefficients, and the results are evaluated through eight information retrieval metrics. Five conclusions are drawn from the findings of this research:

1. The study proposes an automated anomaly detection procedure for existing bridges subjected to different levels of damage. The procedure requires no prior knowledge about the structures to start with, and no operator intervention within the process. Stabilization diagrams and clustering enable reliable modal identification and anomaly detection, despite short-time data segments. For successful automated short-time implementation recognizing at least one pattern for a given segment, the strategy relies on reducible object number in each cluster instead of generating different cluster thresholds for different segments.
2. Modal frequency presents more stable results when compared with the modal damping ratio based on the sharp distinction of damage and undamaged instances. Moreover, although efficient, the basic Boolean operators have limited effects on anomaly detection merge and information retrieval metrics except for very low sigma coefficients. For example, considering Bridge 1’s selectivity parameter for a sigma coefficient equal to 0.5, “and switch” improves the nearest best performer by 19%, whereas “or switch” causes an opposite detection effect. For the sigma coefficients larger than 2, “or switch” converges to frequency-based and “and switch” converges to damping-based detectors. Multivariate anomaly detection can improve the combined performance, however, with a natural sacrifice in computational time.
3. Although frequency-based anomaly detection shows a sharper change from undamaged to damaged states, specific information retrieval metrics favor damping-based anomalies such as selectivity, precision, fall-out, and false discovery rate. A similar conclusion can appear for “and switch” which follows a close pattern in contrast with the “or switch.”
4. Anomaly detection performance and information retrieval metrics depend on the damage level being considered. Metric-scores vs. sigma coefficient curves slide rightwards as the damage definition increases, implying the importance of performance-based criteria to define appropriate thresholds. For example, frequency-based precision and recall curves for low-level damage intersect at a sigma coefficient value of 1.1, whereas they reach 3.4 and 6.6 for medium-level and high-level damages, respectively. This indicates the necessity of monitoring multiple thresholds for a better understanding of the impact of the damaging event.
5. Backwards performance of a seismic anomaly detector can be successfully evaluated with the information retrieval metrics, however, for cases where there is no damaging event for cross-validation, it is untrivial to identify an optimal sigma coefficient. Without any true positives in case of the undamaged bridge, most of the metrics are dysfunctional, leading to an open-ended evaluation criterion: the higher the sigma coefficient, the better. Obviously, determining anomaly detection features under such circumstances is challenging and can be flawed; therefore, monitoring of multiple thresholds together in the same framework is suggested. A similar observation can be made looking at the anomaly time histories, which become more and more insensitive as the sigma coefficient increases.

In summary, with a rigorous system/modal identification, stabilization, clustering, and anomaly detection framework, structural damage can be instantaneously identified with a dependence on damage level being prioritized for alarm. However, referencing a dataset most likely happens with the undamaged initial state, disabling prior determination of anomaly thresholds with the information retrieval metrics. With the widespread usage of anomaly detection based SHM on numerous benchmark tests, backwards analysis of damaged structures can offer more insight on the ideal sigma coefficient values, which motivates the next phases of this research.

## Figures and Tables

**Table 1:** Information retrieval metrics quantifying anomaly detection success/failure rates.

Metric Expectation	Performance According to Detection Threshold σ			
	Decreasing		Increasing	
Favored	Recall or true positive rate	Negative predictive value	Selectivity or true negative rate	Precision or positive predictive value
	$TPR=\frac{TP}{TP+FN}$	$NPV=\frac{TN}{TN+FN}$	$TNR=\frac{TN}{TN+FP}$	$PPV=\frac{TP}{TP+FP}$
Unfavored	Fall-out or false positive rate	False discovery rate	Miss rate or false negative rate	False omission rate
	$FPR=\frac{FP}{FP+TN}$	$FDR=\frac{FP}{FP+TP}$	$FNR=\frac{FN}{FN+TP}$	$FOR=\frac{FN}{FN+TN}$

**Table 2:** Ground motion input and damage details during the test procedure.

Test ID	Description	Time Interval (s)	PGA (g)	Damage
WN1	White noise	0–70	0.07	–
EQ1	Moderate earthquake	70–110	0.33	Bent 1 and Bent 3 yield
WN2	White noise	110–170	0.07	–
EQ2	Severe earthquake	170–210	1.15	Bent 2 yields
WN3	White noise	210–260	0.07	–
EQ3	Extreme earthquake	260–310	1.74	Bent 3 buckles
WN4	White noise	310–379	0.07	–
